# Fast Method for Synthesis of Alkyl and Aryl-*N*-Methylnitrones

**DOI:** 10.3390/molecules16086677

**Published:** 2011-08-08

**Authors:** Serkan Yavuz, Hamdi Ozkan, Naki Colak, Yilmaz Yildirir

**Affiliations:** 1 Chemistry Department, Faculty of Science, Gazi University, Ankara 06500, Turkey; 2 Chemistry Department, Faculty of Science, Çankırı Karatekin University, Çankırı 18100, Turkey; 3 Chemistry Department, Faculty of Science and Arts, Hitit University, Çorum 19030, Turkey

**Keywords:** nitrone, grinding, solid-phase synthesis, solventless

## Abstract

A simple, fast, efficient and eco-friendly procedure was developed for the synthesis of alkyl and aryl-*N*-methylnitrones. The corresponding nitrones of aromatic aldehydes, aliphatic aldehydes and alicyclic carbonyl compounds were prepared from *N*-methylhydroxylamine hydrochloride and Na_2_CO_3_-Na_2_SO_4_ by simply grinding at room temperature without using solvent.

## 1. Introduction

Nitrones are important intermediates in synthetic organic chemistry because they give easily 1,3-dipolar cycloaddition reactions with alkenes, alkynes, isocyanates, isothiocyanates, phosphoranes and sulphenes compounds [1]. Especially, the 1,3-dipolar cycloaddition reactions of nitrone-olefin are interesting due to they can create three new contiguous stereogenic centers in a single step [2,3]. Intermolecular and intramolecular nitrone cycloaddition reactions are also useful methods for the formation of biologically active heterocyclic compounds [4,5,6,7].

A number of methods have been developed for the synthesis of nitrones. Among these, dehydrogenation of *N,N*-disubstituted hydroxylamines, alkylation of oximes and condensation of *N*-methylhydroxylamine hydrochloride with aromatic aldehydes are the most commonly used methods [8]. These methods usually need warming or even refluxing in organic solvents. Moreover these methods mostly suffer from water in which is formed during condensation diminishing the yield of synthesized nitrones. On the other hand, solvent-free reaction procedures have been attracted much more interest for condensation reactions affording near quantitative yields with little or no waste [9,10,11,12,13]. Solvent-free conditions for the synthesis of *α-*aryl-*N*-methylnitrones are generally catalyzed by molecular sieves or silica gel-NaOH, a method first introduced by Bigdeli [14,15]. However, these methods are not successful for the synthesis of the corresponding nitrones of alicyclic and aliphatic carbonyl compounds. Recently, Colacino and co-workers have reported a solvent free synthesis of nitrones by a mechanochemical approach [16]. In that work, nitrone compounds were synthesized after a relatively long reaction time (0.5–2 h) using an automatic ball-milling machine. Herein, we report a simple and solvent-free grinding method for the synthesis of corresponding nitrone compounds of aromatic aldehydes, aliphatic aldehydes and alicyclic ketones by the interaction of carbonyl compounds and *N*-methylhydroxylamine hydrochloride with Na_2_CO_3_-Na_2_SO_4_. Overall conversions were completed in a relatively short time (3–10 min).

## 2. Results and Discussion

The aromatic aldehydes **1a-l** and *N*-methylhydroxylamine hydrochloride were ground at room temperature in the presence of Na_2_CO_3_-Na_2_SO_4_ under solvent-free conditions. In addition, nitrones of aliphatic aldehydes (**3m**, **3n**) and alicyclic ketones (**3o**, **3p**) could also be synthesized by our simple grinding method. 

**Scheme 1:**
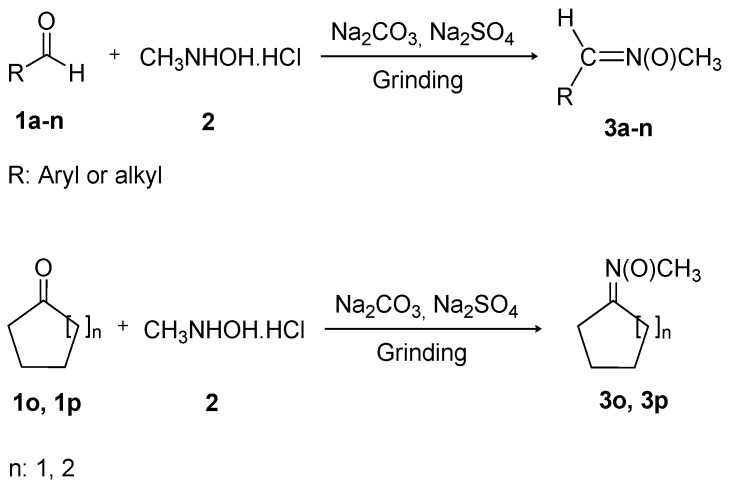
Synthesis of substituted *N*-methyl nitrones.

Aromatic heterocyclic aldehydes and aromatic aldehydes having electron-donating or electron-withdrawing substituents were converted to the corresponding nitrones in high yields in a few minutes. These nitrones were recrystallized from petroleum ether. The condensation reaction between carbonyl compounds and *N*-methylhydroxylamine hydrochloride can also take place in the presence of Na_2_CO_3_. However, the yield of the reaction was lower than yield of the reaction performed in the presence of Na_2_CO_3_-Na_2_SO_4_, because Na_2_SO_4_ absorbed the water formed in the condensation reactions. The substituted *N*-methylnitrones were synthesized as depicted in Scheme 1. Melting points, yields and reaction times of substituted *N*-methyl nitrones are given in Table 1.

## 3. Experimental

All carbonyl compounds, Na_2_CO_3_, Na_2_SO_4_, *N*-methylhydroxylamine hydrochloride and other chemicals were purchased from Merck. All mps were determined in sealed capillaries and are uncorrected. Boiling points were determined in a vacuum distillation apparatus. FT-IR spectra were recorded on a Mattson 1000 spectrometer as KBr pellets or Nujol mulls. ^1^H-NMR spectra were recorded on a Varian Gemini 300 (300 MHz) NMR spectrometer in CDCl_3_. Mass spectra measurements were obtained on a Thermo Finnigan Trace DSQ. MS was performed by means of a Waters ACQUITY UPLC analyser combine with a Micromass LCT Premier TM XE TOF-MS and electrospray ionization (ESI); results agreed satisfactorily with calculated values.

### 3.1. General Method for the Synthesis of Compounds ***3a-l***

A mixture of aromatic aldehyde (10 mmol), *N*-methylhydroxylaminehydrochloride (1.65 g, 20 mmol), sodium carbonate (2.33 g, 22 mmol) and sodium sulphate (0.71 g, 5 mmol) were added in a mortar and ground rapidly with a pestle at room temperature. The reaction was monitored by TLC. Diethylether (10 Ml) was added to the crude product mixture and filtered. The solvent was evaporated under vacuum and the residue was crystallized from petroleum ether.

*C-(5-Methyl-2-thienyl)-N-methylnitrone* (**3g**, C_7_H_9_NOS): Yield: 96%; mp. 90-91 °C; IR (KBr, ν, cm^−1^): 1595 (C=N), 1186 (N→O); ^1^H-NMR (CDCl_3_, δ, ppm): 7.79 (s, 1H, CH), 7.28 (d, *J* = 3.72 Hz, 1H, Ar-H), 6.83 (d, *J* = 3.75 Hz, 1H, Ar-H), 3.88 (s, 3H, NCH_3_), 2.53 (s, 3H, CH_3_); ^13^C-NMR (CDCl_3_, δ, ppm): 15.4, 51.8, 126.0, 130.7, 131.0, 132.0, 144.9; HR-MS (EI): 156.0494 ([M+H]^+^, C_7_H_10_NOS^+^_,_ calc. 156.0483).

*C-(3-Methyl-2-thienyl)-N-methylnitrone* (**3h**, C_7_H_9_NOS): IR (KBr, ν, cm^−1^): 1590 (C=N), 1188 (N→O); ^1^H-NMR (CDCl_3_, δ, ppm): 7.81 (s, 1H, CH), 7.40 (d, *J* = 5.04 Hz, 1H, Ar-H), 6.96 (d, *J* = 5.05 Hz, 1H, Ar-H), 3.92 (s, 3H, NCH_3_), 2.41 (s, 3H, CH_3_); ^13^C-NMR (CDCl_3_, δ, ppm): 14.6, 52.1, 127.7, 129.0, 130.0, 130.5, 140.1; HR-MS (EI): 156.0449 ([M+H]^+^, C_7_H_10_NOS^+^, calc. 156.0483).

*C-(4-Phenyl-2-thienyl)-N-methylnitrone* (**3i**, C_12_H_11_NOS): IR (KBr, ν, cm^−1^): 1598 (C=N), 1185 (N→O); ^1^H-NMR (CDCl_3_, δ, ppm): 7.91 (s, 1H, CH), 7.34-7.70 (m, 7H, Ar-H), 3.92 (s, 3H, NCH_3_); ^13^C-NMR (CDCl_3_, δ, ppm): 40.3, 52.2, 124.5, 127.2, 128.5, 130.0, 131.4, 134.6, 136.0, 142.0; HR-MS (EI): 218.0651 ([M+H]^+^, C_12_H_12_NOS^+^, calc. 218.0640).

*C-(5-Phenyl-2-thienyl)-N-methylnitrone* (**3j**, C_12_H_11_NOS): IR (KBr, ν, cm^−1^): 1590 (C=N), 1183 (N→O); ^1^H-NMR (CDCl_3_, δ, ppm): 7.85 (s, 1H, CH), 7.73 (d, *J* = 1.37 Hz, 1H, thienyl-Ar-H), 7.71 (d, *J* = 1.31 Hz, 1H, thienyl-Ar-H), 7.30-7.70 (m, 5H, phenyl-Ar-H), 3.90 (s, 3H, NCH_3_); ^13^C-NMR (CDCl_3_, δ, ppm): 52.0, 123.6, 127.0, 128.8, 129.8, 131.2, 131.9, 132.4, 135.0, 147.5; HR-MS (EI): 218.0624 ([M+H]^+^, C_12_H_12_NOS^+^, calc. 218.0640).

*C-(5-Methyl-2-furanyl)-N-methylnitrone* (**3l**, C_7_H_9_NO_2_): IR (Nujol, ν, cm^−1^): 1528 (C=N), 1192 (N→O); ^1^H-NMR (CDCl_3,_ δ, ppm): 7.58 (d, *J* = 3.37 Hz, 1H, Ar-H), 7.40 (s, 1H, CH), 6.08 (d, *J* = 3.39 Hz, 1H, Ar-H), 3.72 (s, 3H, NCH_3_), 2.24 (s, 3H, CH_3_); ^13^C-NMR (CDCl_3_, δ, ppm): 13.6, 52.3, 108.7, 116.8, 126.3, 145.2, 154.1; HR-MS (EI): 140.0736 ([M+H]^+^, C_7_H_10_NO_2_^+^, calc. 140.0712).

### 3.2. General Method for the Synthesis of Compounds ***3m-p***

A mixture of carbonyl compound (10 mmol), *N*-methylhydroxylamine hydrochloride (1.65 g, 20 mmol), sodium carbonate (2.33 g, 22 mmol) and sodium sulphate (0.71 g, 5 mmol) were added in a mortar and ground rapidly with a pestle at room temperature. The reaction was monitored by TLC. The 10 mL of diethyl ether was added to the crude product mixture and filtered. After the solvent was evaporated under vacuum and the oily product was taken. The column chromatography was applied to the oily product and purify product was obtained.

*C-(3-Phenylpropylidene)-N-methylnitrone* (**3m**, C_10_H_13_NO): IR (Nujol, ν, cm^−1^): 1561 (C=N), 1176 (N→O); ^1^H-NMR (CDCl_3_, δ, ppm): 7.18-7.36 (m, 5H, Ar-H), 7.05 (t, *J* = 6.41 Hz, 1H, CH), 3.68 (s, 3H, NCH_3_), 2.44-2.84 (m, 4H, CH_2_CH_2_); ^13^C-NMR (CDCl_3_, δ, ppm): 28.4, 33.0, 64.7, 129.1, 129.8, 133.5, 142.3, 154.3; HR-MS (EI): 164.1062 ([M+H]^+^, C_10_H_14_NO^+^_,_ calc. 164.1075).

*C-(2-Phenylpropylidene)-N-methylnitrone* (**3n**, C_10_H_13_NO): IR (Nujol, ν, cm^−1^): 1588 (C=N), 1189 (N→O); ^1^H-NMR (CDCl_3_, δ, ppm): 7.21-7.50 (m, 5H, Ar-H), 6.81 (d, *J* = 7.74 Hz, 1H, CH), 3.65 (s, 3H, NCH_3_), 2.74-2.84 (m, 1H, CH); 1.4 (d, *J* = 7.33 Hz, 3H, CH_3_); ^13^C-NMR (CDCl_3_, δ, ppm): 27.3, 31.9, 62.8, 123.7, 127.0, 136.6, 149.2, 153.7; HR-MS (EI): 164.1099 ([M+H]^+^, C_10_H_14_NO^+^, calc. 164.1075).

## 4. Conclusions

In conclusion, we have developed an environmentally friendly procedure for the synthesis of *N*-methylnitrones in the absence of solvent at room temperature by a grinding method. Na_2_CO_3_-Na_2_SO_4_ was used in this condensation reaction for the first time. In comparison with the classical heating reactions, this procedure has advantageous of milder reaction conditions, shorter reaction times, easier work-up and better yields. In addition, the main advantage of our procedure is to be able to synthesize nitrones of aliphatic aldehydes and alicyclic carbonyl compounds in a solventless medium.

## Figures and Tables

**Table 1:** Reaction time, melting points and yields.

Substrate	Entry	Reaction Time (min)	Products	mp (or bp) (°C)	mp (or bp) (°C) Lit.	Yield (%)
**1a**	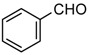	4	**3a**	82–84	82–84 [14]	94
**1b**	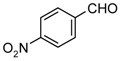	5	**3b**	204–205	210–212 [13]	93
**1c**	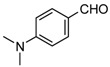	5	**3c**	129–130	129–130 [17]	95
**1d**	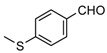	5	**3d**	102–103	103–104 [17]	98
**1e**	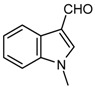	7	**3e**	148–149	148–150 [18]	98
**1f**	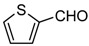	4	**3f**	118–119	118–119 [17]	96
**1g**	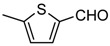	5	**3g**	90–91 ^†^	-	96
**1h**	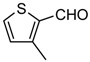	8	**3h**	135–136 ^‡^	-	98
**1i**	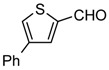	6	**3i**	193–195 ^†^	-	91
**1j**	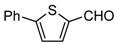	6	**3j**	113–114 ^†^	-	93
**1k**	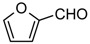	3	**3k**	89–90	89–90 [14]	94
**1l**	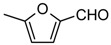	5	**3l**	oil ^†^	-	92
**1m**	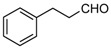	10	**3m**	oil ^‡^	-	62
**1n**	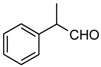	10	**3n**	oil ^‡^	-	76
**1o**	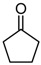	10	**3o**	(72 °C, 0.1 torr)	(70 °C, 0.1 torr) [19]	71
**1p**	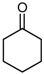	10	**3p**	(88 °C, 0.1 torr)	(80 °C, 0.1 torr) [19]	68

All compounds are characterized on the basis of IR, ^1^H-NMR, ^13^C-NMR and HR-MS spectral data. ^†^ Compounds not found in the literature. Their spectral data are given in the Experimental section. ^‡^ Compound found in the literature, but melting (or boiling) points and spectral data not found. Their spectral data are given in the Experimental section.
